# Testing hypotheses of pteraspid heterostracan feeding using computational fluid dynamics

**DOI:** 10.1080/02724634.2023.2272974

**Published:** 2023-12-19

**Authors:** Madleen Grohganz, Humberto G. Ferrón, Zerina Johanson, Philip C. J. Donoghue

**Affiliations:** 1 Palaeobiology Research Group, School of Earth Sciences, University of Bristol, Life Sciences Building, Tyndall Avenue, Bristol BS8 1TQ, U.K., madleen.grohganz@bristol.ac.uk; humberto.ferron@bristol.ac.uk; phil.donoghue@bristol.ac.uk; 2 Natural History Museum, Cromwell Road, London SW7 5BD, U.K., z.johanson@nhm.ac.uk

## Abstract

The ecological context of early vertebrate evolution has been characterized as a gradual shift from passive to more active feeding modes. This evolutionary scenario has been based largely on poorly constrained inferences of the feeding ecology of extinct stem-gnathostomes, among which heterostracans are the earliest. Pteraspidiform heterostracans possessed a feeding apparatus composed of rod-like oral plates with rows of rostrally facing denticles, previously interpreted as an adaptation for suspension feeding. Here, we test this hypothesis using computational fluid dynamics. We simulate water flow around 2D models consisting of rows of denticles both rostrally facing and reversed, to assess whether these orientations create recirculation patterns that are a hydrodynamic adaptation to suspension feeding. All tested models, independent of denticle orientation, show similar flow, velocity, and vorticity patterns. Recirculation patterns, highest velocity, and vorticity develop directly on top of the denticles and in spaces between the denticles. Therefore, we reject the hypothesis that denticle orientation is an adaptation for recirculation linked to suspension feeding. The denticles may instead have served to prevent material from lodging between the plates.

## INTRODUCTION

**FIGURE 1.**
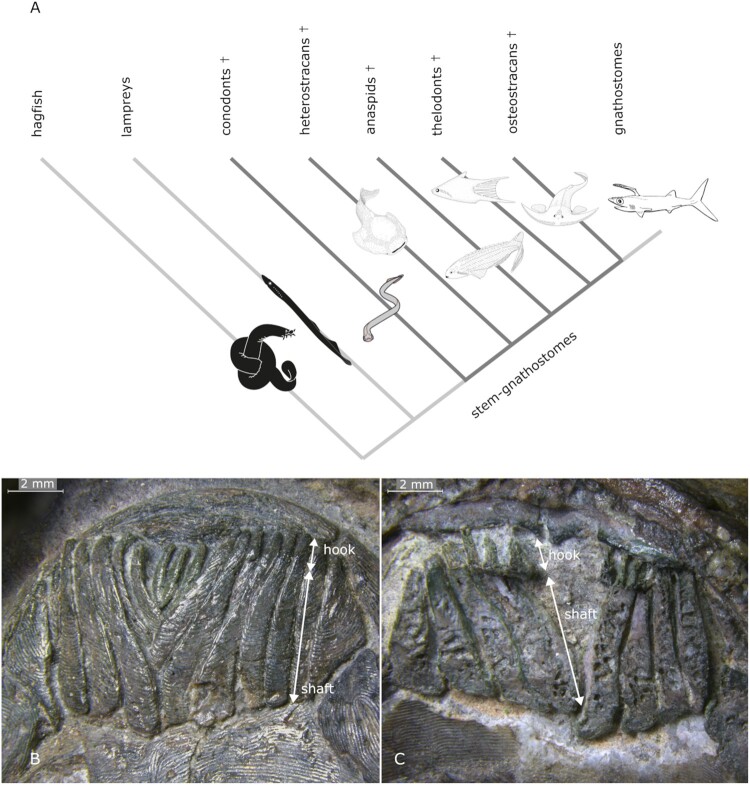
**A**, diversity and relationships of jawless and jawed vertebrates, crosses indicate extinct clades; Articulated, V-shaped oral plate apparatus of Protopteraspis vogti; **B**, aboral; **C**, oral views. Rostral is to the top of the image in **B** and **C**.

**FIGURE 2.**
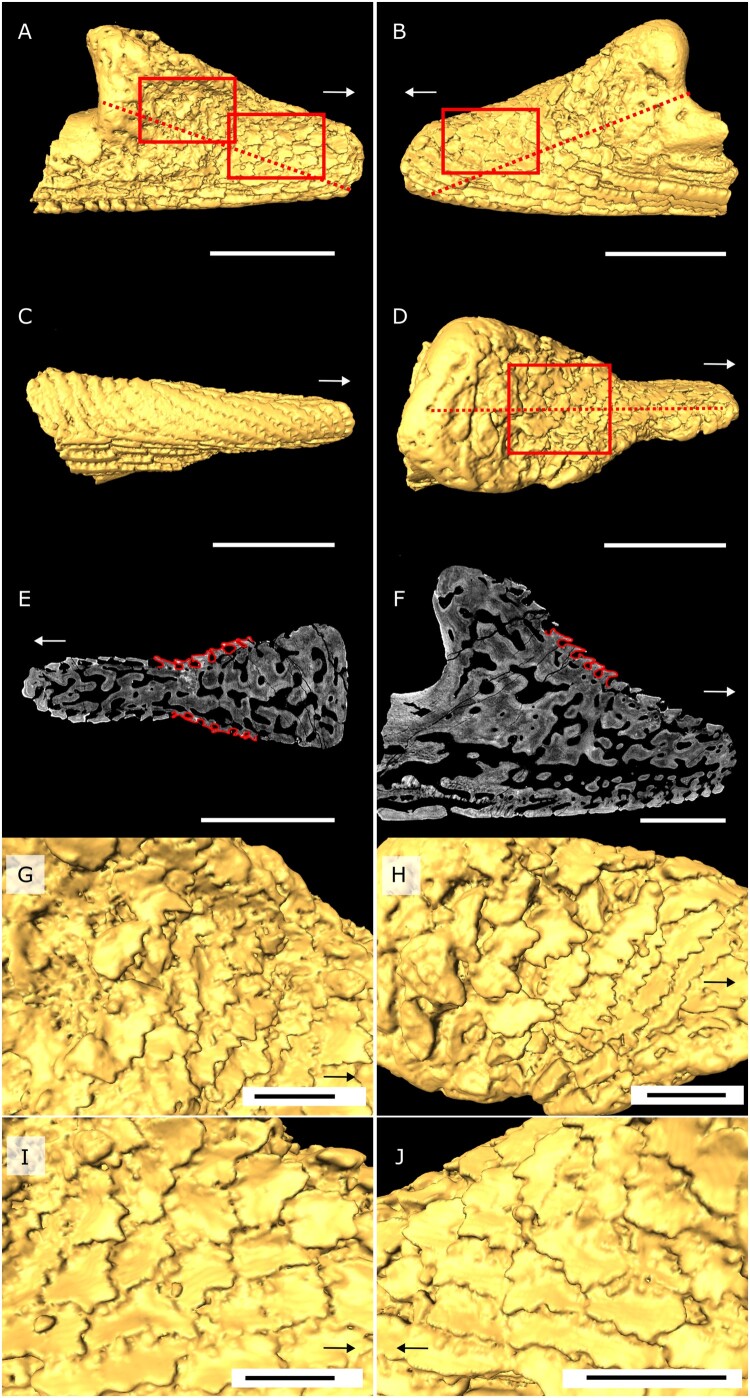
Oral plate of *Loricopteraspis dairydinglensis* (specimen NHMUK PV P43710; Lower Devonian, Ditton Group, Dairy Dingle, near Neenton, Shropshire, U.K.); **A** and **B**, 3D surface model lateral views of the distal hook, dashed line indicates cutting plane (see **E**), boxes indicate positions of close-ups of rostrally facing denticles (see **G**, **I**, and **J**); **C**, 3D surface model aboral view; **D**, 3D surface model oral view, dashed line indicates cutting plane (see **F**), box indicates position of close-ups of rostrally facing denticles; **E**, virtual section through the hook perpendicular to denticles (see **A** and **B**) (showing denticles on lateral oral plate surfaces, which outlines act as a basis for the CFD denticle model); **F**, virtual section through ridge of the oral plate hook (see dashed line in **D**) (showing denticles on oral surface, which outlines act as a basis for the CFD denticle model); **G**, close-up of denticles on lateral side (see left box in **A**); **H**, close-up of denticles on oral side (see box in **D**); **I**, close-up of denticles on lateral side (see right box in **A**); **J**, close-up of denticles on lateral side (see box in **B**). Arrows indicate rostral direction. Scale bars equal 1000 microns (**A**–**E**); 500 microns (**F**); 300 microns (**G**–**J**).

The New Head (Gans & Northcutt, 1983) and New Mouth (Mallatt, 1996) hypotheses argue that early vertebrate evolution is characterized by a long-term trend from suspension feeding (more passively removing particulate food items trapped by some means of filtration) to successively more active predatory lifestyles in the jawed vertebrates. Living jawless vertebrates include scavengers and parasites in the adults but the feeding ecology of extinct jawless and jawed stem-gnathostomes (which are phylogenetic intermediates of living jawless and jawed vertebrates) remains unclear. Heterostracans are among the earliest stem-gnathostomes, both phylogenetically and stratigraphically (Blieck, 1984; Donoghue et al., 2000; Halstead, 1973; Keating et al., 2015) (Fig. 1A); their feeding ecology is disputed. The ventral margin of the pteraspidiform heterostracan mouth was occluded by a series of small, narrow, rod-like oral plates, organized in a V-shaped arrangement (Purnell, 2002; Stensiö, 1964; White, 1935) (Fig. 1B, C) and of unclear function. The lateral surfaces of their distal hooks are covered with rows of rostrally facing triangular or maple-leaf-shaped sharply pointed denticles (Purnell, 2002) (see Fig. 2G). These denticles are similar in size and shape to denticles in the nasal passages of some shark species, the function of which is also unclear (Reif, 1985).

Early studies (Kiaer, 1928) interpreted the oral plates of the heterostracan *Pteraspis* as used for predation, actively biting or crushing prey, analogous to gnathostome jaws. Arguments against an active predatory feeding mode have mostly been based on proposed functional constraints due to the lack of jaws in heterostracans (Halstead, 1973; Romer, 1959, 1966, 1970) though this view is belied by macrophagy in jawless lampreys, hagfish, and conodonts (Gans & Northcutt, 1983; Purnell, 1995). A scavenging feeding mode has been proposed, with the oral plates similar in arrangement and function to the hagfish feeding apparatus (Janvier, 1974; Jarvik, 1980; Stensiö, 1932). The mouth of *Pteraspis* has also been interpreted as protrusible, forming a scoop-like structure used for detritus feeding (White, 1935).

Purnell (2002) investigated the competing hypotheses of heterostracan feeding, demonstrating that denticles directed rostrally, out of the mouth would have prevented effective intake of food rather than aiding in prey apprehension. Purnell (2002) argued that these denticles would have been readily damaged and broken when encountering sediment or prey in a deposit feeding or predation scenario; wear occurs on the oral plates, but only on the caudo-ventral surface as well as on the ventral side of the headshield. Purnell (2002) interpreted this as evidence for a nektobenthic lifestyle with heterostracans abrading their ventral surface when close to the seafloor. However, he argued that this observation could not be reconciled with the oral plates being used to actively bite or sediment-scoop, as no wear was observed on the distal tips of the oral plates, nor on the denticles themselves. In addition, Purnell (2002) noted fusion of some of the oral plates, which would have prevented the lower margin of the mouth from opening and spreading out as a sediment scoop. Combining these observations led him to preclude a mechanical function, leaving only the suspension feeding hypothesis. However, Purnell (2002) did not specify a mechanism of suspension feeding, based either on oral plates acting as suspension feeding organs, or on suspension feeding organs associated with the gills.

Testing the suspension feeding hypothesis in general remains challenging but it is necessary to progress our understanding of early vertebrate feeding ecology (Janvier, 1996). Detailed information on the internal anatomy of the oral cavity and structures potentially involved in suspension feeding such as the gill arches or gill pouches is scarce and ambiguous. Evidence of internal anatomy has been limited to impressions on the dermal skeleton, with no direct evidence for associated filtering structures (Halstead, 1973; Kiaer, 1932). Based on the lack of evidence of internal filtering structures most authors have focused on the oral plates and their potential role in suspension feeding in heterostracans. While Purnell (2002) was not able to resolve the function of the rostrally facing denticles, his results have been reinterpreted to indicate that the rostrally facing denticles were involved in filter-feeding (Janvier, 2015; Lingham-Soliar, 2014; Stiefel, 2021). Our goal is to test this specific hypothesis, that the rostrally facing structures on the oral plates are an adaptation to suspension feeding.

Living suspension feeding fishes use different filter feeding mechanisms. One potential mechanism for heterostracans is similar to vortical cross-step filtration. A vortical cross-step mechanism has been described for living suspension feeding fishes, which filter particles out of the water while avoiding clogging of their filter structures (Brooks et al., 2018; Sanderson et al., 2016). Flow passes parallel or tangential to the filter structure, which generates vortices. This is a well-described physical principle, that vortices tend to form behind structures acting as steps in the presence of crossflow (Biswas et al., 2004). The vortices clear particles from the filter structure and help with suspension feeding by suspending and transporting particles backwards in between the steps (Brooks et al., 2018; Sanderson et al., 2016).

In living filter feeding fish, the filtrate leaves between the gills, which are unknown in heterostracans beyond impressions in the headshield, mentioned above. However, the physical principle of vortex generation behind “steps” remains relevant to heterostracans in which flow would have occurred parallel to the denticles of the oral plates. If the rostrally facing denticles (oriented against the direction of flow) on the oral plates are an adaptation to suspension feeding (Janvier, 2015; Lingham-Soliar, 2014; Stiefel, 2021), vortices should emerge in between the denticles (acting as steps). The vortices should develop between the individual rows of denticles, in the open, flask-shaped spaces. The emerging flow patterns would suspend food particles and assist in suspension feeding in heterostracans similar to what has been modeled for recent suspension feeding fish using cross-step filtration. Critically, if the rostrally facing denticle orientation is an adaptation to suspension feeding, these features should not occur when denticles are oriented in the direction of flow (i.e., caudally facing). We adopt the caudally facing orientation as our null model.

Here, we use Computational Fluid Dynamics (CFD) to test this specific aspect of the suspension feeding hypothesis. CFD is a technique that simulates fluid flow and its interaction with solid surfaces in a virtual environment. It is a valuable tool in paleobiological studies, providing insights into feeding mode and locomotion of extinct organisms (e.g., Cracknell et al., 2021; Ferrón et al., 2020; Gibson et al., 2019; Gutarra et al., 2019; Rahman et al., 2015; Rigby & Tabor, 2006; Shiino et al., 2012) since it requires few (if any) biological assumptions, simply characterizing the relationship between physical objects and fluid flow. In our experiments, we simulated the interaction of fluid flow with 2D models of heterostracan oral plate denticles and visualized flow patterns, velocity, and vorticity (a vector field, that gives a microscopic measure of the local rotation at any point in the fluid, the tendency for elements of the fluid to “spin” around a certain axis).

## MATERIALS AND METHODS

### Model Construction

Our study focused on analysis of the oral plates in *Loricopteraspis dairydinglensis* from the Lower Devonian, Ditton Group, of Dairy Dingle, near Neenton, Shropshire, U.K. This is the same taxon (and we analyze some of the same specimens) in which Purnell (2002) demonstrated the presence of rostrally facing denticles on the lateral surfaces of the hook component of the oral plates; Purnell (2002) illustrated similar denticles in *Protopteraspis* and *Rhinopteraspis. Loricopteraspis* is one of very few taxa for which oral plates, isolated from the matrix and well-preserved, are available. This combination of factors makes *Loricopteraspis dairydinglensis* an ideal model for testing hypotheses of feeding in heterostracans. Furthermore, its oral plates are considered a representative feature of pteraspidiform heterostracans, rendering conclusions derived from *L. dairydinglensis* generally relevant (Randle et al., 2022).

**FIGURE 3.**
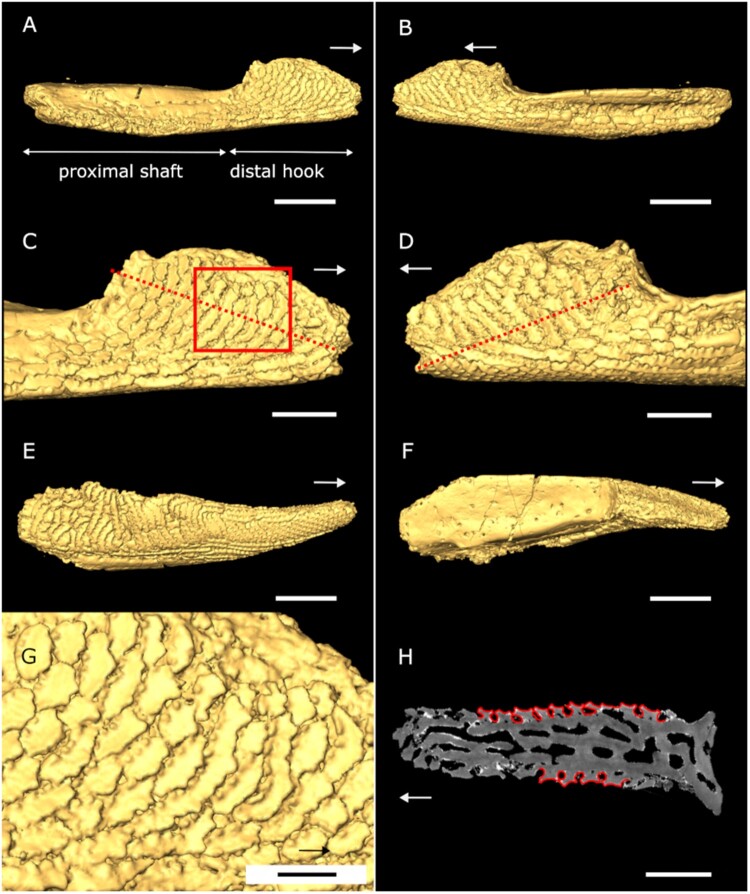
Oral plate of *Loricopteraspis dairydinglensis* (specimen NHMUK PV P43713; Lower Devonian, Ditton Group, Dairy Dingle, near Neenton, Shropshire, U.K.); **A** and **B**, 3D surface model lateral views; **C** and **D**, 3D surface model of distal hook in lateral views, dashed line indicates cutting plane (see **H**), box indicates position of close-up of rostrally facing denticles (see **G**); **E**, 3D surface model aboral view; **F**, 3D surface model oral view; **G**, close-up of denticles on lateral sides (see box in **C**); **H**, virtual section through oral plate hook perpendicular to denticles (see dashed line in **C** and **D**) (showing denticles on lateral oral plate surfaces, which outlines act as a basis for the CFD denticle model). Arrows indicate rostral direction. Scale bars equal 1000 microns (**A**, **B**, **E**, **F**); 500 microns (**C**, **D**); 200 microns (**G**); 400 microns (**H**).

**FIGURE 4.**
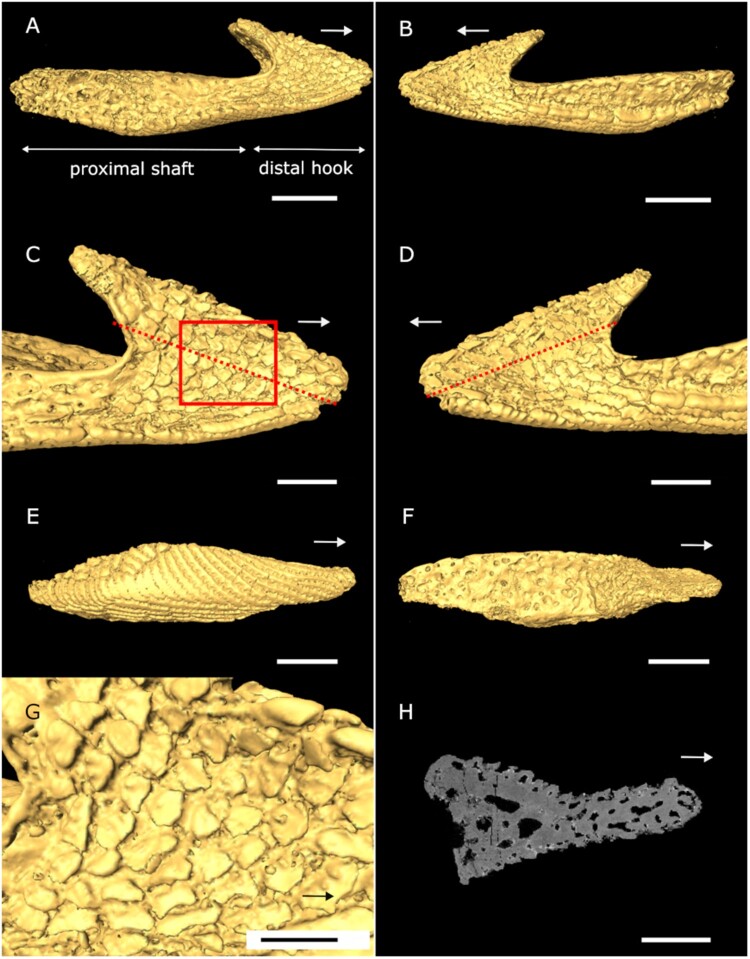
Oral plate of *Loricopteraspis dairydinglensis* (specimen NHMUK PV P43711; Lower Devonian, Ditton Group, Dairy Dingle, near Neenton, Shropshire, U.K.); **A** and **B**, 3D surface model lateral views; **C** and **D**, 3D surface model of distal hook in lateral views, dashed line indicates cutting plane (see **H**), box indicates position of close-up of rostrally facing denticles (see **G**); **E**, 3D surface model aboral view; **F**, 3D surface model oral view; **G**, close-up of denticles on lateral sides (see box in **C**); **H**, virtual section through oral plate hook perpendicular to denticles (see dashed line in **C** and **D**). Arrows indicate rostral direction. Scale bars equal 1000 microns (**A**, **B**, **E**, **F**); 500 microns (**C**, **D**, **H**); 400 microns (**G**).

The oral plates were characterized tomographically using Synchrotron Radiation X-ray Tomographic Microscopy (srXTM; Donoghue et al., 2006) (Figs. 2–4) (see S1, Supplementary Data 1 for further details). Virtual sections were created in Avizo Lite (Version 9.5.0, https://www.fei.com/software/avizo3d) perpendicular to the denticles on the lateral sides of the plates. Measurements include denticle height, width and space between the denticles, to inform the models. Two sets of 2D models, idealized denticle and empirical denticle, were created as binary tiff images in Inkscape (Version 1.0.1) consisting of a row of denticles (around 20) sitting on a flat surface (S2, Supplementary Data 1) (model length L_idealized_ = 0.0025 m, L_empirical_ = 0.00235 m). The idealized model captures generalized denticle shape, while the empirical model additionally accounts for shape variation depending on where the denticles are cut (midline or more lateral position). Models with caudally facing denticles were created by mirroring the respective model with rostrally facing denticles. All the models were converted to step files in Rhinoceros 3D (Version 7, https://www.rhino3d.com) using the ‘Vectorize’ function for further processing and analysis. Surface area measurements were taken in Inkscape with the ‘Measure path’ function based on images of the oral plates obtained from Avizo 3D surface models.

We did not model the entire oral plate as our focus is on testing the effect of the orientation of the denticles, which are located on the lateral sides of the distal-most oral plate tips (see Figs. 2–4). The denticles are arranged in parallel rows, through which we took perpendicular cross sections (see dashed lines in Figs. 2–4). With this approach we capture the variability in shape of the denticles and the spaces between the different rows, which justifies 2D modeling. We take a 2D approach because our principal interest is in the orientation of the denticles relative to the current, i.e., whether the denticles are directed against the current or in direction of flow, which is the case for dermal denticles elsewhere on the body. 3D modeling of several denticle rows is unlikely to provide additional material insight germane to the hypothesis that the rostrally facing denticle orientation is an adaptation to suspension feeding. A 3D approach could enhance our understanding of fluid flow relative to denticle orientation but is neither necessary nor desirable for our purposes.

### Computational Fluid Dynamics

Simulations of water flow around 2D denticle models were performed in ANSYS-Fluent 2020 R1 Academic (www.ansys.com). To set up a sufficiently large domain and allow the flow to fully develop around the model, we followed recommendations in the literature (Rahman, 2017). The computational domain was set to a 2D rectangle, 0.0575 m in length (at least 3 times the total length L upstream plus at least 20 times the total length L downstream) and around 0.01 m in height (at least 20 times the domain baseline-sample midline height V1) (Supplementary Data 1: S3). The model was fixed to the lower surface of the computational domain. A normal inflow velocity boundary condition was assigned to one end of the domain and an outlet with zero pressure to the opposite. The upper surface of the computational domain was set to “open,” slip symmetry boundary conditions, whereas the lower surface and the model-fluid interface were set as a “solid,” non-slip boundary, fixing fluid velocity at zero.

The domain was meshed using the triangle element method with increasing element size with increasing distance from the object. Generally, default mesh size parameters were used except for computational domain element size (10^−4^ m) and refinement domain (body sizing) element size (10^−5^ m). We performed independence tests to ensure that results are independent of changing domain and mesh parameters. These tests were repeated for different inlet velocities, 0.05 m/s and 0.5 m/s. A solution was considered independent if the converged value for drag force did not change by more than 5% between a simulation and the next. The analyses show that results are independent of the domain and mesh parameters chosen (Supplementary Data 2).

Simulations were run using a 2D, incompressible, viscous laminar flow model and a stationary solver to compute the steady-state flow patterns. We chose a laminar flow model, because our denticle models are mm scale, which results in Reynolds numbers far below the boundary for turbulent flow (Re_turbulent _> 2000) (see values calculated below). This assumption is further justified by the fact that the oral plate denticles are positioned at the rostral-most margin of the mouth, supporting that in life they would encounter an undisturbed incoming flow.

Fluid properties of water were assigned using the ANSYS property database. CFD analyses were performed with inlet velocities of 0.05 m/s (Reynolds number Re = 125 for the idealized models, Re = 117.5 for the empirical models) and 0.5 m/s (Re = 1250 for the idealized models, Re = 1175 for the empirical models). The different inlet velocities reflect swimming speeds of modern fish from more passive to more active swimmers (Froese & Pauly, 2021). Swimming speeds for heterostracans have recently been estimated based on ancestral character state reconstruction (Ferrón & Donoghue, 2022). Pteraspidomorphs show relatively low reconstructed swimming speeds of 1.13 ± 0.10 body lengths per second. Considering the body length of taxa with rostrally facing denticles on the hooks of their oral plates (*Loricopteraspis*, *Protopteraspis*, and *Rhinopteraspis*), which is in the centimeter to decimeter range, these estimates match well with our chosen inlet velocities.

**Figure 5.**
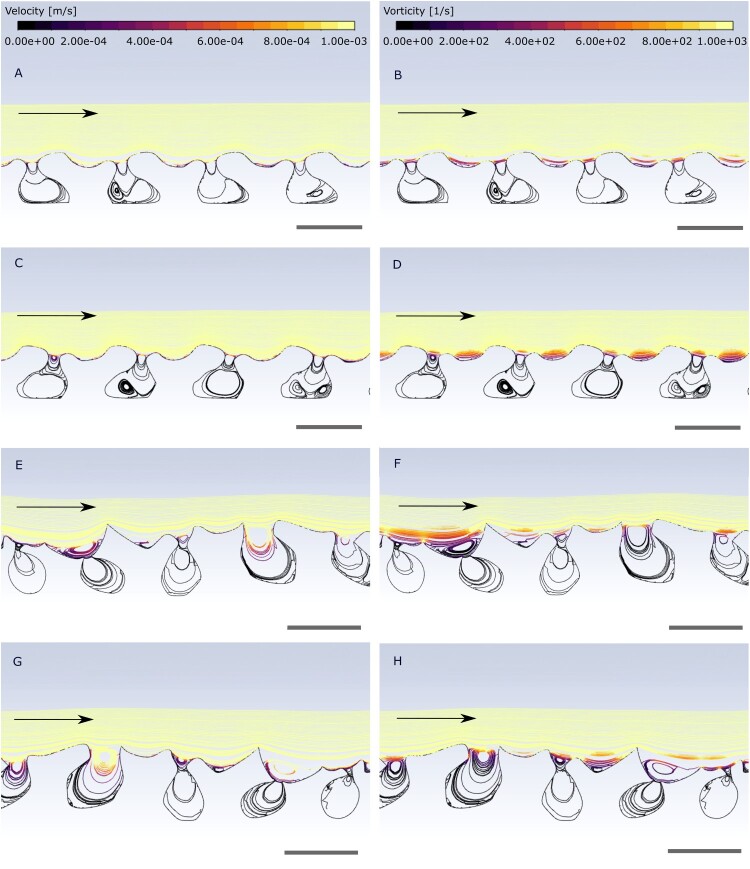
Pathline velocity and vorticity plots of different tested models, inlet velocity 0.5 m/s. **A**, idealized rostrally facing denticle model, velocity; **B**, idealized rostrally facing denticle model, vorticity; **C**, idealized caudally facing denticle model, velocity; **D**, idealized caudally facing denticle model, vorticity; **E**, empirical rostrally facing denticle model, velocity; **F**, empirical rostrally facing denticle model, vorticity; **G**, empirical caudally facing denticle model, velocity; **H**, empirical caudally facing denticle model, vorticity. Scale bar equals 100 microns.

We assume ram filter feeding with a unidirectional flow for heterostracans (Maisey, 1996; Mark-Kurik, 1992), as the jawless nature of heterostracans would strongly constrain the amount heterostracans could open their mouths. This drastically reduces pumping capacity necessary for pump filter feeding and bidirectional flow. For pump filter feeding an oscillating pump with pharyngeal arches and strong muscles is needed to generate currents (Drenner et al., 1982; Sanderson et al., 1993). However, what little is currently known about the internal anatomy of heterostracans is only inferred from impressions on the internal surface of the dermal skeleton and reliable evidence for the soft body anatomy necessary for pump filter feeding is missing (Halstead, 1973; Kiaer, 1932). All results were visualized as pathline plots of velocity and vorticity over the surface of the denticle models. The first and last 5–10 denticles of each model were excluded from the visualization to avoid the influence of edge effects. We describe the results of the experiments based on an inlet velocity of 0.5 m/s (see Fig. 5).

**Institutional Abbreviations**—**NHMUK**, Natural History Museum, London, U.K.

## RESULTS

### General Morphology of the Oral Plates and Morphological Variation of Rostrally Facing Denticles

The pteraspidiform heterostracan oral plates follow a general morphology consisting of two principal domains, the distal hook and the proximal shaft (see Figs. 1, 3, and 4; positional descriptors refer to the point of articulation of the oral plates with the post-oral plate with proximal being close to the point of articulation). This has been shown for *Pteraspis* (Ball & Dineley, 1961; Kiaer, 1928; White, 1935), *Protopteraspis* (Heintz, 1962), *Loricopteraspis* (White, 1961), and *Rhinopteraspis* (Tarlo, 1961). Those taxa also show the rostrally facing denticles on their lateral hook surfaces, for which a suspension feeding mode has been proposed. On their aboral surface, the *Loricopteraspis* oral plates are covered with rows of interlocking ridges, which have serrated edges on both sides (Figs. 2C, 3E, and 4E). The lateral and oral surfaces of the hook are covered with denticles (Fig. 2G–J, 3G, and 4G).

The denticles show a range of morphological variation across different specimens. In the specimen described by Purnell (2002) (NHMUK PV P43710), the denticles occur on the lateral and oral surfaces of the distal hook and appear discrete (see Fig. 2G–J). However, this one specimen is unrepresentative of the range of denticle morphology seen in a broader sample of oral plates within the same collection. The denticles on the lateral surfaces of the hook in NHMUK PV P43713 are fused into ridges (Fig. 3G). On oral plate NHMUK PV P43711 the rostrally facing denticles on the lateral surfaces of the hook are more discrete (resembling the denticles described by Purnell 2002) but they are abraded and have lost their pointed edges (Fig. 4G). To account for this morphological variation, we took virtual cross sections through the discrete as well as ridged denticles from the different oral plate specimens. These cross sections clearly reflect the morphology of our empirical denticle model (see Figs. 2E, F and 3H) on which we performed our CFD experiments.

### CFD Analyses

In the case of rostrally facing denticle models, for both the idealized and empirical models, highest velocities in the order of magnitude of 10^−4^ m/s (roughly correlated with approximate denticle length, ca. 110 microns) can be observed on top of the individual denticles and in the upper part of the spaces between the denticles (Fig. 5A, E). Velocity values range between 2×10^−4^ m/s and 8×10^−4^ m/s. Vorticity magnitude also shows highest values, in the order of magnitude of 10 ^+ 2^ 1/s, in the same positions (Fig. 5B, F). Vorticity values range between 2×10 ^+ 2^ 1/s and 8×10 ^+ 2^ 1/s. Velocity and vorticity patterns in the empirical models are not as constrained as in the symmetrical idealized models. In the empirical models (Fig. 5E, F) the velocity and vorticity patterns reach higher up into the undisturbed laminar flow above the denticles and the spaces between them, especially where the denticles show a bigger difference in size.

For the caudally facing denticle models, both idealized and empirical, results are similar, with respect to magnitude of highest velocity and location above and between the denticles (Fig. 5C, G). A similar vorticity magnitude pattern also occurs (Fig. 5D, H).

The pathlines of all models, independent of the denticle orientation, show patterns of recirculation in the spaces between the individual denticles as well as in the concavities directly on top of the denticles (Fig. 5). The flow further above the denticles remains undisturbed. Generally, idealized and empirical models exhibit overall comparable flow, velocity, and vorticity patterns.

For an inlet velocity of 0.05 m/s, absolute values for velocity and vorticity are generally smaller, but the overall flow, velocity, and vorticity patterns strongly resemble those described for an inlet velocity of 0.5 m/s, for both the idealized and empirical models (Supplementary Data 1: S4). Highest velocities in the order of magnitude 10^−5^ m/s are observed on top of the individual denticles and in the upper part of the spaces between the denticles (Supplementary Data 1: S4 A, S4 C, S4 E, S4 G). Highest values for vorticity magnitude, in the order of magnitude 10 ^+ 1^ 1/s, show similar positions (Supplementary Data 1: S4 B, S4 D, S4 F, S4 H). Patterns of recirculation in the spaces between the individual denticles and directly on top of the denticles can be observed in the pathlines of all models, independent of the denticle orientation. The flow above the denticles remains undisturbed.

## DISCUSSION

In living groups, suspension feeding involves removal of water-suspended particles via some type of biological or physical filtering mechanism associated with filtering structures such as gill arches (Hamann & Blanke, 2022; Hentschel & Shimeta, 2008; Jørgensen, 1966; La Barbera, 1984). In the case of heterostracans, suspension feeding was proposed after the exclusion of alternative feeding modes (Purnell, 2002). We have no knowledge of heterostracan gill arches, but the presence of oral denticles has been used as evidence for a filter-feeding function (Janvier, 2015; Lingham-Soliar, 2014; Stiefel, 2021). Hence, we tested whether the rostrally facing structures would have created vortices between them, serving to dislodge and re-suspend food particles within the ingested water comparable to living suspension feeding fish using cross-step filtration (Brooks et al., 2018; Sanderson et al., 2016).

Another filter feeding mechanism, that has recently been described in manta rays, is ricochet separation (Divi et al., 2018). This mechanism utilizes rostrally facing as well as caudally facing structures for filtration, which bear a superficial resemblance to heterostracan denticles. Particles are ricocheted off these filtering structures by vortices to become concentrated in the esophagus while the filtrate passes through without clogging the filter. Even though the filtering structures might superficially resemble heterostracan denticles and their orientation relative to the water flow, this system is probably not a suitable comparison, as the degree to which heterostracans could open their mouth relative to manta rays was probably restricted due to lacking jaws. The mouth of *Pteraspis* has been interpreted as protrusible (White, 1935), but Purnell (2002) suggested the oral plates were partially fused, contradicting this. Thus, a ricochet filtration mechanism would potentially divert most of the particles away from the mouth as they bounce off the filtering structures outside the mouth, which would hinder effective feeding. Therefore, if heterostracans were suspension feeders, cross-step filtration would be the most likely mechanism. Our analyses show patterns of recirculation, highest velocity, and vorticity in the upper space between the oral denticles and directly on top. The observed patterns all occur at a small-scale, while flow further above the denticles remains undisturbed. These patterns are observed in all models with rostrally as well as caudally facing denticle orientation, independent of model type (idealized or empirical) and inlet velocity (0.5 m/s or 0.05 m/s). Our original hypothesis predicted that, if rostrally facing denticles are an adaptation to suspension feeding, these recirculation, velocity, and vorticity patterns would only occur in models with rostrally facing denticles. Our results allow us to reject this hypothesis, but not our null model. Thus, we conclude that the rostrally facing structures of heterostracan oral plates are not an adaptation to suspension feeding.

What function then, if any, did the rostrally facing denticles serve? There are no direct modern analogs of rostrally facing denticles on feeding structures. However, similar denticles have been observed in the oropharyngeal region of non-suspension feeding sharks (most of them caudally facing, oriented with their cusps pointing towards the esophagus). In most shark groups these denticles are arranged in a broadly spaced pattern, but Sphyrnidae show the densest arrangement most closely resembling the denticle patterns observed in the heterostracan oral plate denticles (Reif, 1985). Several possible functions of shark oral denticles have been discussed in the literature, but no conclusion has been reached so far. Nelson (1970) discusses the pharyngeal denticles in sharks as “pharyngeal pads” analogous to the consolidated upper and lower pharyngeal tooth plates of osteichthyans, which might function in moving food items from the pharynx into the esophagus. Reif (1985) proposed that the denticles could protect the skin against damage by food particles. In addition, oral denticles are found circularly arranged around oral papillae, presumably for protection or for directing water flow around the taste buds (Atkinson & Collin, 2012). The caudally facing denticles could also decrease drag of water flow through the oral cavity and consequently improve ram ventilation (Reif, 1978) or prevent parasites from attaching in the oral cavity (Raschi & Tabit, 1992). In heterostracans the denticles are rostrally facing, which would have impeded food transport or drag reduction (Purnell, 2002). There is no evidence for taste buds or skin associated with the oral plate denticles. The heterostracan denticles occur on lateral faces of the distal extremity of the oral plates, constituting no more than ∼15% of the overall surface area. There the oral plates are thought to have been exposed in life without a soft tissue cover at their distal tips where they are narrowest and are envisaged to have been separate from one another during function (White, 1935). As such, the role of the denticulated surfaces may have been to prevent material, oversized food particles, sediment particles, or parasites, from passing in between the oral plates, clogging the spaces between them and preventing occlusion.

While we have been able to test and reject the hypothesis that the rostrally directed structures on the oral plates of heterostracans are an adaptation to suspension feeding, the nature of heterostracan feeding remains an open question. Most of these feeding hypotheses are based on morphological comparisons with extant jawed vertebrates or with extant jawless fish, which have specialized feeding modes. They propose a mechanical function either related to active biting or crushing (Kiaer, 1928), scavenging (Janvier, 1974; Jarvik, 1980; Stensiö, 1932), herbivorous macrophagy (Bendix-Almgreen, 1986), or detritus feeding (White, 1935).

These appear to be rejected based on an absence of wear on the surface of the denticles of the oral plates, which is interpreted to preclude a mechanical food-processing function (Purnell, 2002). Moreover, definitive tests of mechanical functional hypotheses will remain out of reach in the absence of knowledge of heterostracan oral anatomy, including the relative arrangement of the oral plates in vivo and constraints on their movement. This is not intractable; articulated heterostracans are known from museum collections representing different collapse orientations (e.g., *Errivaspis*; White, 1935) that may be used to reconstruct the original three-dimensional geometry of the oral plates (Briggs & Williams, 1981). Combined with advances in digital characterization and modeling of fossil materials (Cunningham et al., 2014) it should prove possible to construct digital models of the heterostracan oral apparatus for use in computational functional testing. Detailed knowledge of the feeding mode of heterostracans and other stem-gnathostomes gained from these analyses is crucial to testing macroevolutionary scenarios such as the New Head and New Mouth hypotheses and elucidating early vertebrate evolution.

## Supplementary Material

Supplemental Material

## Data Availability

Data are available at the University of Bristol data repository, data.bris, at https://doi.org/10.5523/bris.1maxt1atfbrht23rm3qyy1nujr

## SUPPLEMENTARY FILES

Supplementary Data 1.docx: S1, details on srXTM methodology; S2, 2D denticle models idealized and empirical, scale bar 500 microns; S3, dimensions of computational domain and refinement domain; S4, pathline velocity and vorticity plots of different tested models, inlet velocity 0.05 m/s.

Supplementary Data 2.xlsx: Results of independence analyses for inlet velocity 0.5 m/s and inlet velocity 0.05 m/s.
