# Supplementary material for: Testing hypotheses of pteraspid heterostracan feeding using computational fluid dynamics
Source: J Vertebr Paleontol. 2023 Dec 19;43(2):e2272974. doi: 10.1080/02724634.2023.2272974 (PMC11969682; doi:10.1080/02724634.2023.2272974)
Supplement: Supplemental Material [file UJVP_A_2272974_SM9497.zip › Updated_Supplementary File 1_R3docx.docx]

Journal of Vertebrate Paleontology (JVP)

Testing hypotheses of pteraspid heterostracan feeding using computational fluid dynamics

MADLEEN GROHGANZ,^*,1^, HUMBERTO G. FERRON,^2,†^, ZERINA JOHANSON,^3^, and PHILIP C.J. DONOGHUE,^*,4^

^1^Palaeobiology Research Group, School of Earth Sciences, University of Bristol, Life Sciences Building, Tyndall Avenue, Bristol BS8 1TQ, United Kingdom, [madleen.grohganz@bristol.ac.uk](mailto:madleen.grohganz@bristol.ac.uk);

^2^Palaeobiology Research Group, School of Earth Sciences, University of Bristol, Life Sciences Building, Tyndall Avenue, Bristol BS8 1TQ, United Kingdom, [humberto.ferron@bristol.ac.uk](mailto:humberto.ferron@bristol.ac.uk);

^3^Natural History Museum, Cromwell Road, London SW7 5BD, United Kingdom, [z.johanson@nhm.ac.uk](mailto:z.johanson@nhm.ac.uk);

^4^Palaeobiology Research Group, School of Earth Sciences, University of Bristol, Life Sciences Building, Tyndall Avenue, Bristol BS8 1TQ, United Kingdom, [phil.donoghue@bristol.ac.uk](mailto:phil.donoghue@bristol.ac.uk)

S1: Details on srXTM methodology

srXTM investigations were performed at the X02DA TOMCAT beamline of the Swiss Light Source, Paul Scherrer Institute (Villigen, Switzerland) following a standard acquisition approach with the rotation axis located in the middle of the field of view and the acquisition of 1501 projections equiangularly distributed over 180° of rotation. Scan details are as follows: for specimen NHMUK PV P43710, 4× objective with exposure time of 60 ms at 20 keV and resulting isotropic voxel dimensions of 1.625 μm; for specimen NHMUK PV P43711, 4× objective with exposure time of 300 ms at 21 keV and resulting isotropic voxel dimensions of 1.625 μm; for specimen NHMUK PV P43713, 4x objective with exposure time of 50 ms at 16 keV and resulting isotropic voxel dimensions of 1.625 μm. Projections were post-processed and rearranged into flat- and dark-field-corrected sinograms, and reconstruction was performed on a 60-core Linux PC farm. Slice data derived from the scans were analysed and manipulated using Avizo software for computed tomography, at the University of Bristol.

S2: 2D denticle models idealized and empirical, scale bar 500 microns


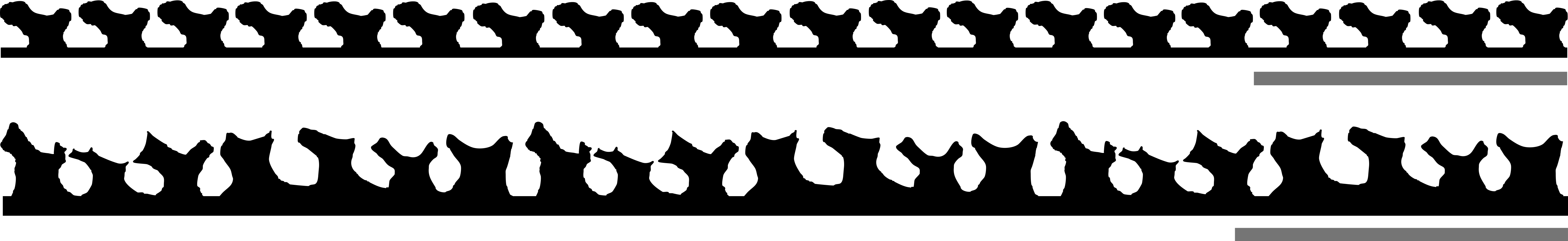


S3: Dimensions of computational domain and refinement domain


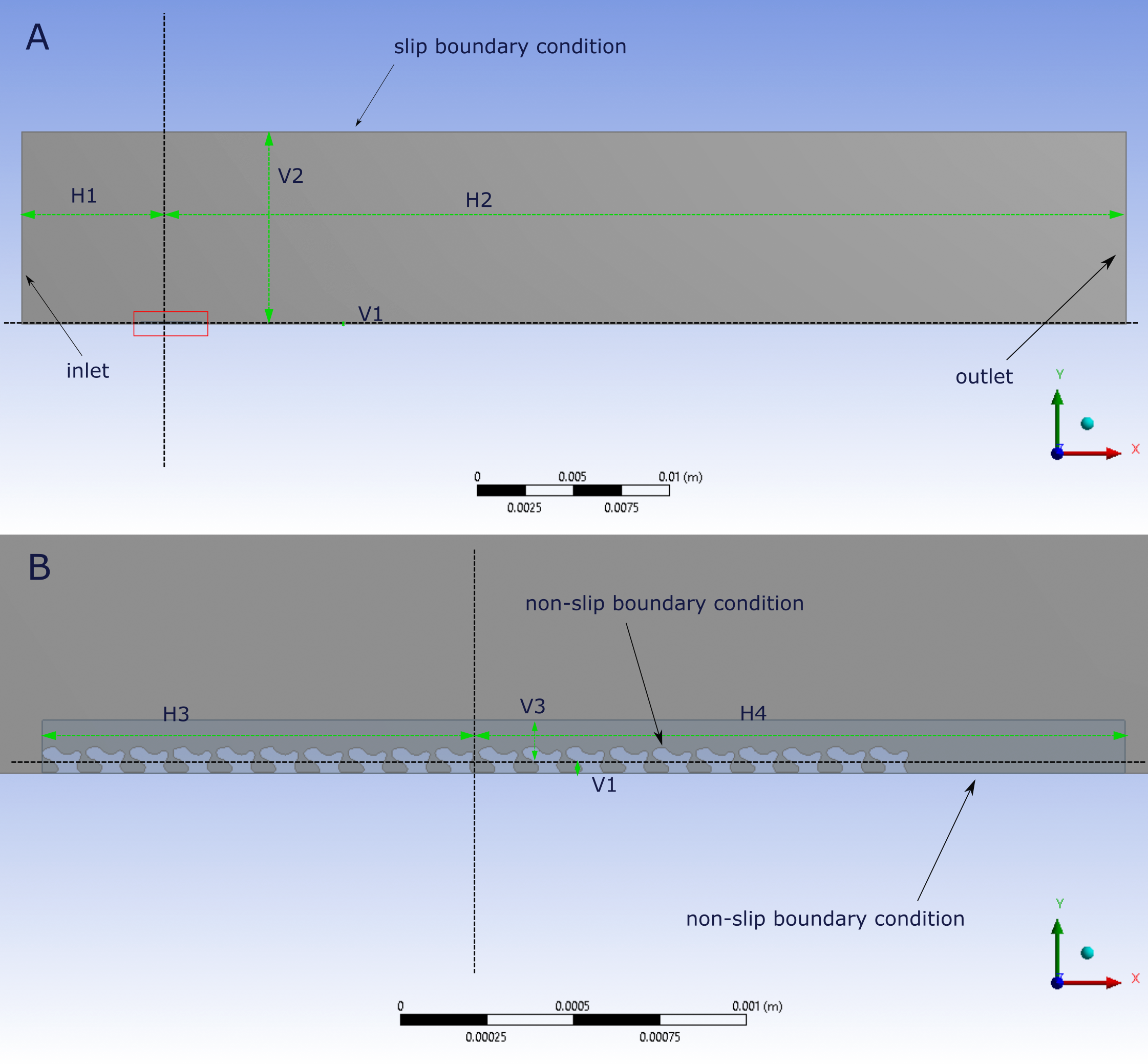


(A) dimensions of computational domain: upstream domain length H1= 0.0075 m, downstream domain length H2= 0.05 m, domain baseline-sample midline height V1= 0.000032 m (idealized models)/0.000042 m (empirical models) (see also V1 in (B)), domain height V2= 0.01 m, red box illustrates the position of the refinement domain shown in (B); (B) dimensions of refinement domain: upstream refinement domain length H3= 0.00125 m, downstream refinement domain length H4= 0.001875 m, refinement domain height V3= 0.00012 m. Flow is simulated from the left to the right in all cases (inlet to outlet).

S4: Pathline velocity and vorticity plots of different tested models, inlet velocity 0.05 m/s


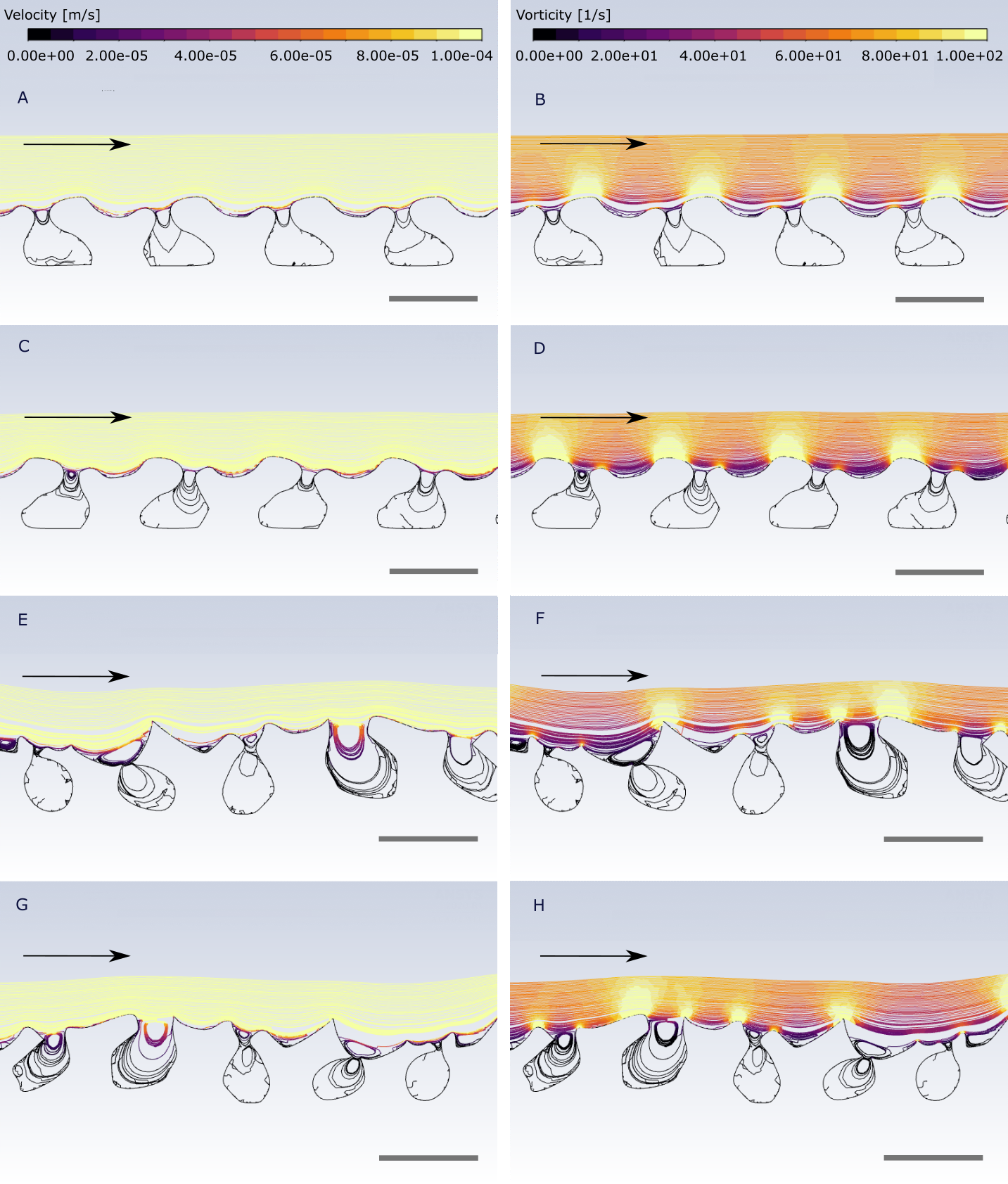


(A) idealized rostrally-facing denticle model, velocity, (B) idealized rostrally-facing denticle model, vorticity, (C) idealized caudally-facing denticle model, velocity, (D) idealized caudally-facing denticle model, vorticity, (E) empirical rostrally-facing denticle model, velocity, (F) empirical rostrally-facing denticle model, vorticity, (G) empirical caudally-facing denticle model, velocity, (H) empirical caudally-facing denticle model, vorticity. Scale bar 100 microns.
